# Ultrastructure of in vitro Matured Human Oocytes

**DOI:** 10.5812/ircmj.7379

**Published:** 2013-12-05

**Authors:** Abbas Shahedi, Mohammad Ali Khalili, Mehrdad Soleimani, Shekoufeh Morshedizad

**Affiliations:** 1 Department of Anatomy, Yazd Branch, Islamic Azad University, Yazd, IR Iran; 2 Yazd Institute for Reproductive Sciences, Shahid Sadoughi University of Medical Sciences, Yazd, IR Iran; 3 Islamic Azad University, Yazd, IR Iran

**Keywords:** In vitro Maturation, Germinal Vesicle Oocytes, Metaphase-I Oocytes, Ultrastructure

## Abstract

**Background:**

Approximately 20% of recovered oocytes are immature and discarded in intracytoplasmic sperm injection (ICSI) procedures. These oocytes represent a potential resource for both clinical and basic science application.

**Objective:**

The aim of this study was to evaluate the ultrastructure architecture of in vitro matured human oocytes using transmission electron microscopy (TEM).

**Materials and Methods:**

A total of 204 immature oocytes from infertile patients who underwent ICSI cycles were included in this prospective study. Immature oocytes were divided into two groups: (i) GV oocytes (n = 101); and (ii) MI oocytes (n = 103). Supernumerary fresh in vivo matured oocytes (n = 10) were used as control.

**Results:**

The rates of maturations were 61.38% for GV and 73.78% for MI oocytes in IVM medium (P = 0.07). However, the rate of oocyte arrest was significant between groups (P <0 .05). Ultrastructurally; in vitro and in vivo matured oocytes appeared round, with a homogeneous cytoplasm, an intact oolemma and an intact zona pellucida. However, immature oocytes indicated numerous large mitochondria-vesicle complexes (M-VC).

**Conclusions:**

Ultrastructural changes of M-VC in IVM groups emphasize the need for further research in order to refine culture conditions and improve the implantation rate of in-vitro matured oocytes.

## 1. Background

Approximately 20% of aspirated oocytes following hormone stimulation are immature at the germinal vesicle (GV) or metaphase I (MI) stage. These oocytes are discarded due to their reduced potential for embryo development under current culture conditions (1). However, this cohort of oocytes is benefit for studies aimed at elucidating the mechanisms of in vitro maturation of human oocytes and might ultimately contribute to the pool of embryos available for embryo transfer (2, 3). It would be clinically useful if the in-vitro maturation rates of these oocytes could be improved because some patients produce only a few oocytes, while others may produce mostly immature oocytes (4).

Although, healthy infants have been born following IVM protocols. but, the pregnancy rates are significantly lower when compared with in vivo matured oocytes in gonadotrophin stimulated cycles (5). Thus, improving the IVM success rate remains an important challenge to obtain better efficiency for fertility preservation (6). IVM has several advantages to reduce costs; avoiding the side effects of ovarian hyperstimulation syndrome and simplifying treatment for certain infertile couples (7). The ability to resume meiosis is independent of the cumulus cells (8). Thus, the oocyte is able to undergo nuclear maturation without presence of cumulus and granulosa cells. Clinical practice has shown a correlation between morphological quality of IVM oocytes and fertilization, cleavage and implantation rates. Routine evaluation of oocyte morphology by phase-contrast microscopy (PCM) is an important predictive marker of oocyte quality, currently utilized for evaluation of the success of a given ART program (9). However, low-resolution morphological assessment is not always a sufficient measure of oocyte fertility potential and developmental competence (10). Therefore, use of electron microscopy, integrated by other investigational approaches, seems especially effective in evaluation of oocyte quality.

## 2. Objectives

The present study was therefore aimed to investigate the ultrastructure of immature human oocytes obtained from ICSI cycles that were matured in vitro with transmission electron microscopy (TEM).

## 3. Materials and Methods

### 3.1. Study Population

This prospective study included 85 women aged 28-40 years who were consecutively treated with ICSI in Yazd institute for reproductive sciences. Informed consent was obtained from each patient before the start of research. Supernumerary MII oocytes from cases that were cancelled due to azoospermia on the day of oocyte retrieval were used for TEM. Also, oocytes that were distinguished to be at M-I or GV stages on day 0 were assigned for IVM.

Ovarian stimulation was induced with long protocols using GnRH agonist down-regulation, followed by rFSH (Gonal-F; Serono, Switzerland). The ovarian response was controlled by transvaginal ultrasound and serum estradiol concentration on the day of hCG injection. When diameter of at least two follicles was larger than 18 mm, 10,000 IU of hCG (i.m.; Profasi, Serono) was administered. Oocyte pick-up was performed 36 h after hCG injection under transvaginal ultrasound-guidance. All follicles larger than 14 mm were subjected to pick-up, while follicles smaller than 14 mm were not punctured. There was uniformity of the ultrasound measurements between our physicians (11). After oocyte retrieval, the oocytes were denuded by a short exposure with 80 IU/ml hyaluronidase (Sigma Co, St. Louis, Mo, USA) and mechanically by using fine-bore glass pipettes. After 2-3 h, the oocytes were then assessed for nuclear maturation (GV, MI or MII), under the stereo microscope (Olympus Co, Tokyo, Japan) and separated based on maturity. Those that were considered mature underwent ICSI. This study was approved by the ethical committee of the institute.

### 3.2. Groups

Group 1: The supernumerary mature oocytes were obtained from consenting ICSI patients (n = 10). Groups 2 and 3 were cases with immature oocytes at GV (n = 101) and MI stage (n = 103), respectively. Immature oocytes in groups 2 and 3 were transferred to pre-warmed IVM medium (3 oocytes per 50µl droplet under mineral oil) containing Hams F10 (Biochrom Co, Germany) supplemented with 0.75 IU LH, 0.75 IU FSH (Ferring Co, Germany) with 40% human follicular fluid (HFF). The oocytes were then cultured in a 5% CO2 incubator at 37 °C with high humidity. After 36 h culture, the oocyte maturation was determined by the presence of the first polar body.

### 3.3. Electron Microscopy

A total of 30 mature oocytes were prepared for TEM. From these oocytes, ten oocytes were matured in-vivo and the rest were matured by IVM technology. Oocytes were fixed and processed for TEM as described by Nottola et al. (12). Oocytes were fixed in 1.5% glutaraldehyde (Sigma, Mo, USA) in 0.1 M phosphate buffer for 2-5 days at 4 °C, and washed in the same buffer for 10 min, and post-fixed in 1% osmium tetroxide (Agar Scientific, Stansted, UK) in phosphate buffer, and washed again in the same buffer. Oocytes were then embedded in small blocks of 1% agar (Sigma, Mo, USA), dehydrated in increasing concentrations of ethanol, immerssion in propylene oxide for solvent substitution and individually embedded in Araldite resin (Merk, Germany). The PH of both fixatives ranged from 7.2 to 7.4 at room temperature. For light microscopy, the oocytes were sectioned at a thickness ranging from 0.5 to 1 µm and stained with Toluidine blue, and then examined by light microscopy (Zeiss Axioskop). Ultrathin sections (60 - 80 nm) were cut and stained with uranyl acetate (7 min) and lead citrate (13 min). These sections were observed and photographed with a TEM at 80KV (Zeiss, Germany).

### 3.4. Statistical Analysis

Differences in oocyte maturational stages, between the groups were calculated and compared using Chi-square test. P < 0.05 was considered statistically significant. Calculations were performed by SPSS software (version 16, USA).

## 4. Results

### 4.1. Oocyte Maturation

61.38% of GV and 73.78% of M-I stage oocytes were matured after 36h in IVM medium. There were significant differences in rates of immature oocytes arrest between two groups (GV = 21.78%, M-I = 10.67%, P < 0.05).

### 4.2. Ultrastructure of M-II Oocyte

**Figure 1.**
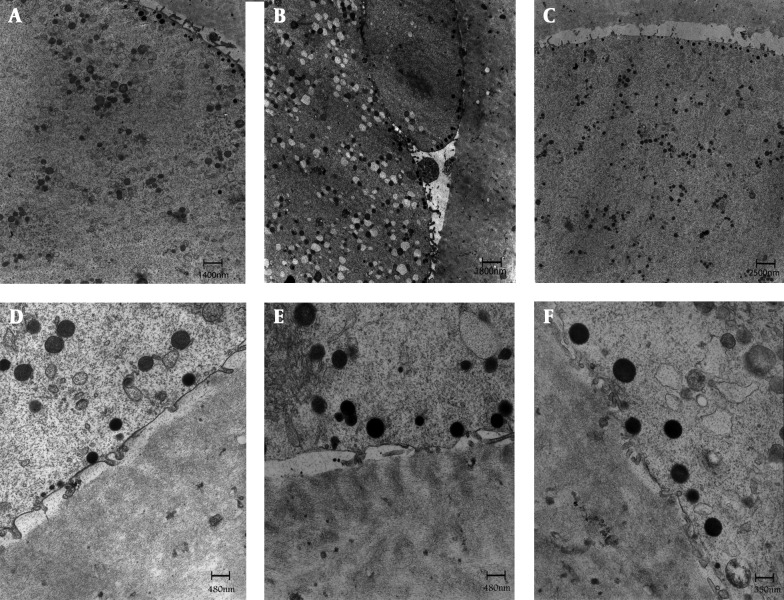
General Fine Structure and Organelle Microtopography are shown by Transmission Electron Microscopy. In control (A, D), GV (B, E) & MI (C, F) stage oocyte after IVM. Microvilli are numerous and long on the oolemma of oocytes. A rim of electron-dense cortical granules (arrows) is seen just beneath the oolemma of oocytes. ZP = zona pellucida; mv = microvilli; CG = cortical granules; O = oocyte; PVS = perivitelline space; PB = polar body

In the control M-II oocytes, the zona pellucida (ZP) consisted of closely packed electron dense fibrilar material. The perivitelline space (PVS) was uniform and scarce debris was usually found in it. The oolemma was continuous, and provided with numerous long-thin microvilli were uniformly distributed on the oolemma, except in the zone of polar body extrusion where microvilli were absent. Another feature was the presence of round cortical granules with an electron dense appearance that was located just beneath the oolemma (Figures 1A and D). 

**Figure 2.**
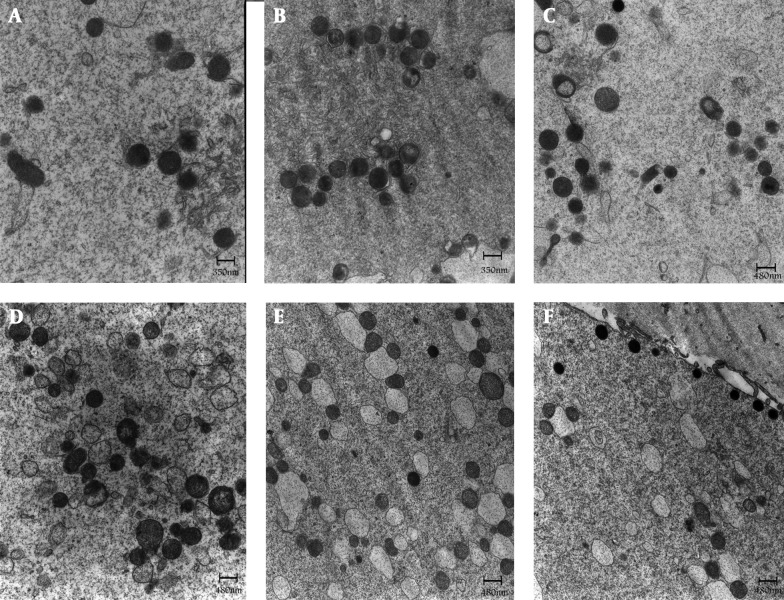
Control Oocyte (A, D), GV (B, E) & MI (C, F) Stage Oocyte after IVM. Mitochondria are generally rounded and provided with few peripheral or transverse cristae. Dumbbell shaped, possibly dividing mitochondria can be occasionally find in the ooplasm (C). Migrating cortical granule is seen in C (arrow). Voluminous aggregates between mitochondria and elements of smooth endoplasmic reticulum are seen (A-C). Note the presence of complexes between mitochondria and vesicles of smooth endoplasmic reticulum in D - F (arrows). Migrating cortical granules are seen in E (arrow heads). SER = smooth endoplasmic reticulum; M = mitochondria

The majority of the oocyte organelles consisted of aggregates of smooth endoplasmic reticulum (SER) surrounded by spherical or elliptical shaped mitochondria (M-SER aggregates). Small and large, spherical M-SER aggregates were found randomly distributed in the ooplasm (Figure 2A). Small mitochondria-vesicle complexes (M-VC) were also observed (Figure 2D). The arc-like or transverse cristae of mitochondria were irregularly placed on the periphery and parallel to the outer mitochondrial membrane. These cristae contained a moderately electron dense matrix (Figures 2A and D). 

### 4.3. Ultrastructure of GV and MI Oocytes after IVM

The ultra-structural characteristics of IVM oocytes had some similarity to that of the control M-II oocytes. However, the most notable feature of the matured oocytes after IVM was the presence of numerous large M-VC within their cytoplasm as compared to in-vivo matured oocytes (Figures 2E and F). In general, the population of M-VC was more distributed in GV when compared with MI oocytes (Figure 2E). Small and large, spherical M-SER aggregates were found randomly distributed in the ooplasm (Figure 2B and C). Other organelles, such as mitochondria, cortical granules, microvilli, PVS and ZP were similar between groups (Figures 1E and F, 2B and C). 

## 5. Discussion

In ovarian stimulation cycles, maturation of 85-90% oocytes may be triggered by the administration of hCG 36 h before oocyte retrieval. In conventional IVF cycles, the remaining immature oocytes at the time of collection are not suitable for ICSI. These immature oocytes, upon patient’s agreement, are generally destined to research (5). Why these oocytes fail to complete nuclear maturation remains speculative. It has been proposed that the follicles may be resistant or less responsive to hormonal stimulation (13) or they simply may be at an earlier developmental phase (14). In this study, we demonstrated that rate of maturation of GV oocytes was lower than M-I oocytes (61.38% vs. 73.78%). This is, probably due to the differences at the oocyte developmental stage at the time of oocyte collection. Previous studies indicated the IVM rate as low as 37% for GV oocytes collected in stimulated cycles (5, 14). These results are not comparable with our findings (61.38%), which is likely due to the differences at duration of maturation and composition of IVM medium. Previous studies, investigating the clinical outcome of immature oocytes retrieved from stimulated ICSI cycles, have shown that early matured oocytes yield significantly higher fertilization and developmental potential than their later matured counterparts (15). Tan et al. (4) reported the maturation rate of 70.8% for human M-I oocytes collected from stimulated cycles, which is similar to our findings (1). Despite the fact that implantation rate per transferred embryo using in-vitro matured oocytes doesn’t yet reach the outcomes of conventional IVF (16), this technique is already offered to patients at high risk of developing hyper-stimulation syndrome or for fertility preservation. In vivo and within primary oocytes, cellular components of endoplasmic reticulum (ER), mitochondria and Golgi complexes are seen around the nucleus (17). Once, meiosis starts, organelles distribute throughout the cell. As reported, no ultra-structural differences could be observed between spontaneously ovulated and ovulation-induced oocytes (18). In this study, numerous large M-VC observed in the IVM oocytes that may be sign of prolonged stay in vitro (in vitro aging) and could be potential determinants of a reduced oocyte competence for fertilization. M-SER aggregates are currently considered as a precursor of mitochondria-vesicle complexes (M-VC) and may play a role in the production of materials useful at fertilization and / or in rapid neoformation of membranes during early embryogenesis (19). M-SER aggregates may also regulate local concentration of free calcium and ATP production, thus acting on different cellular activities including mediation of calcium–dependent signal transduction pathways at fertilization (20). The ultra-structural alterations in the arrangement of M-SER aggregates could be, among other factors, potential determinants of a reduced oocyte competence for fertilization, possibly due to disturbances in calcium homeostasis. Variations in shape and size of M-SER, without ultra-structural changes in both SER tubules and mitochondria, could be related to different morphodynamic stages of these elements (12).

In our study, cortical granules were normal and no ultra-structural changes were demonstrated between control and IVM groups. Wessel et al. (21) reported that cortical granules are Golgi-derived; membrane bound spherical or slightly ellipsoid organelles formed during the early stages of oocyte and at maturation (21). Exocytosis of the cortical granule content into PVS immediately after oocyte fertilization lead to changing of the ZP glycoproteins, establishing the block to polyspermy. This study showed that in all groups, oocytes were surrounded by an intact, continuous oolemma, provided with long microvilli projecting into a PVS with regular shape and width. It has been reported that proper distribution of microvilli on the oocyte surface is determinant for the spermatozoon-oocyte fusion (22).

In conclusions, immature human oocytes at different stages of development subjected to the IVM showed minimum of alteration in cytoarchitecture. However, ultra structural changes of M-VC following IVM emphasize the need for further research in order to refine culture conditions and improve the implantation rate of in vitro matured oocytes.
